# A potent nonporphyrin class of photodynamic therapeutic agent: cellular localisation, cytotoxic potential and influence of hypoxia

**DOI:** 10.1038/sj.bjc.6602527

**Published:** 2005-04-19

**Authors:** W M Gallagher, L T Allen, C O'Shea, T Kenna, M Hall, A Gorman, J Killoran, D F O'Shea

**Affiliations:** 1 Department of Pharmacology, Centre for Synthesis and Chemical Biology, Conway Institute of Biomolecular and Biomedical Research, University College Dublin, Belfield, Dublin 4, Ireland; 2 Department of Chemistry, Centre for Synthesis and Chemical Biology, Conway Institute of Biomolecular and Biomedical Research, University College Dublin, Belfield, Dublin 4, Ireland

**Keywords:** photodynamic therapeutic agent, cellular localisation, cytotoxic potential

## Abstract

We have developed a totally new class of nonporphyrin photodynamic therapeutic agents with a specific focus on two lead candidates azadipyrromethene (ADPM)01 and ADPM06. Confocal laser scanning microscopy imaging showed that these compounds are exclusively localised to the cytosolic compartment, with specific accumulation in the endoplasmic reticulum and to a lesser extent in the mitochondria. Light-induced toxicity assays, carried out over a broad range of human tumour cell lines, displayed EC_50_ values in the micro-molar range for ADPM01 and nano-molar range for ADPM06, with no discernable activity bias for a specific cell type. Strikingly, the more active agent, ADPM06, even retained significant activity under hypoxic conditions. Both photosensitisers showed low to nondeterminable dark toxicity. Flow cytometric analysis revealed that ADPM01 and ADPM06 were highly effective at inducing apoptosis as a mode of cell death. The photophysical and biological characteristics of these PDT agents suggest that they have potential for the development of new anticancer therapeutics.

Photodynamic therapy (PDT) is an emerging technique for the treatment of cancer and noncancerous conditions (Sharman *et al*, 1999; Dolmans *et al*, 2003). It involves the administration of a photosensitiser, followed by its excitation with low-energy tissue-penetrating light. The interaction of the photosensitiser and light results in photochemical production of activated oxygen species that attack key structural entities within the targeted cells. The most important of these oxygen species is widely believed to be singlet oxygen (^1^O_2_) (Weishaupt *et al*, 1976). ^1^O_2_ has a short half-life (0.6 × 10^−6^ s) and an approximate diffusion distance of 0.1 *μ*m in aqueous media (Moan and Berg, 1991). As such, PDT has the potential to be a highly localised and selective form of cancer therapy, since only irradiated areas are affected, provided the photosensitiser is nontoxic in the absence of light.

Reactive singlet oxygen is capable of causing direct tumour cell killing, vascular shutdown and local damage by recruitment of inflammatory and immune mediators (Henderson and Dougherty, 1992). The efficacy of PDT is influenced by several factors including the type of photosensitiser, its intracellular localisation, dose of photosensitiser, light dose and fluence rate, oxygen availability and time between administration of the photosensitiser and light exposure (Dolmans *et al*, 2003). PDT can result in either apoptotic or necrotic cell death, with this appearing related to the specific subcellular localisation of the PDT agent (Noodt *et al*, 1996, 1999).

Photofrin was the first and, at the moment, remains the most common, clinically used PDT agent (MacDonald and Dougherty, 2001). It has been approved for use in the United States, Canada and Japan for the treatment of oesophageal, endobroncheal, bladder, lung, stomach, cervical and skin cancers (Dolmans *et al*, 2003). Despite its clinical use, Photofrin is not an ideal photosensitiser, as it is an undefined mixture of dimeric and oligomeric compounds derived from the acid treatment of haematoporphyrin. Many attempts to develop other photosensitisers have primarily focused on poly-pyrrole macrocycles, such as porphyrins and phthalocyanines.

The investigation of non-porphyrin photosensitisers for the development of novel PDT agents has been considerably less extensive (Wainwright, 1996). Development of such agents may identify compounds with improved efficacy, reduced side-effects and increased possibilities for modification. In this context, we have developed a family of novel PDT agents, the BF_2_-chelated tetraaryl-azadipyrromethenes (ADPM). We have previously demonstrated that the ADPM class of compounds display excellent photochemical and photophysical properties (Gorman *et al*, 2004). Moreover, the synthesis of these compounds is straightforward and results in defined single compounds with the ability to optimise key photophysical characteristics. Herein, we examine in detail the biological activity of two members of this family of photosensitisers, ADPM01 and ADPM06 (Figure 1A).

## MATERIALS AND METHODS

### Cells

All cell lines unless otherwise stated were obtained from the American Type Culture Collection. HeLa, MRC5, HT-1080, MDA-MB-231, MCF-7 and T47-D cells were cultured in minimum essential medium (MEM) supplemented with 10% (v v^−1^) foetal calf serum (FCS), 1% (v v^−1^) non-essential amino acids, 50 U ml^−1^ penicillin, 50 *μ*g ml^−1^ streptomycin, 20 mM HEPES and 1% (v v^−1^) L-glutamine (Gibco-BRL). SW480, SW620 (obtained from Professor D Croke, Royal College of Surgeons, Dublin), A2780 and A2780cp70 cells (Obtained from Professor R Brown, Cancer Research UK, Beatson Laboratories, Glasgow) were cultured in RPMI-1640 medium plus the above-mentioned supplements. All cells were maintained in 5% CO_2_ (v v^−1^) and 21% O_2_ (v v^−1^) at 37°C unless otherwise stated.

### Hypoxia

Cells were exposed to low oxygen conditions in a hypoxia chamber (Coy Labs). Exponentially growing cells plus media were equilibrated in the hypoxia chamber for 24 h. The hypoxia chamber consisted of an airtight glove box, with the atmosphere continuously monitored by an oxygen analyser. An oxygen concentration of 1% was maintained throughout the chamber, with the balance comprised of 5% CO_2_, nitrogen and water vapour from a humidified chamber.

### Photosensitising agents

The synthesis of the ADPM family of compounds has been described previously (Gorman *et al*, 2004). The photosensitiser (1.3 × 10^−5^ mol) was dissolved in 2.0 ml of dry THF. A pre-prepared mixture of Cremophor EL (CrEL)/1,2-propanediol (10 : 3 v v^−1^) (0.2 ml) was added to the solution and placed in a sonic bath, under N_2_, for 1 h. The THF was removed under reduced pressure and the remaining mixture was slowly dissolved in phosphate-buffered saline (PBS) solution (15 ml). This solution was then passed through an Acrodisc 25 mm syringe filter (with 0.2 *μ*m HT Tuffryn membrane) and transferred to a volumetric flask where the solution was made up to 25 ml with PBS solution. Dilutions into MEM containing 10% (v v^−1^) FCS were used for assaying. The exact concentration of the photosensitiser was confirmed by UV–Visible spectral analysis of the solutions prior to assaying. The quantity of CrEL/1,2-propanediol in an assayed photosensitiser solution was always less than 0.03%.

### Uptake and clearance studies

Cells were seeded at 30 000 cells well^−1^ in chamber slides and allowed to adhere for 24 h. For the uptake studies, ADPM01 (1 × 10^−5^ M) was then added to each chamber and incubated at 37°C in the dark. Following appropriate incubation periods (5 min–6 h), the cells were washed four times with media to remove all free photosensitisers. The cells were then fixed with 3.7% formaldehyde/PBS for 15 min at 37°C, after which the cells were washed twice with PBS. Mounting fluid (Vectashield) and a coverslip were then placed on each slide. For the clearance studies, the cells were incubated at 37°C with ADPM01 (1 × 10^−5^ M) for 3 h in the dark. In this case, the cells were fixed at various time points (15 min – 24 h) after removal of the drug. The slides were then viewed using an Axio fluorescent microscope (Zeiss). A DAPI filter (Band pass 356) was used to accurately count the number of cells in each field of view and a rhodamine filter (Long pass 590) was used to quantify fluorescence from ADPM01. LabWorks version 4.0 (Bioimaging Systems) was used to calculate the average fluorescence intensity of ADPM01 per cell.

### Confocal laser-scanning microscopy

Cells, grown on eight-well chamber slides (Nunc), were incubated in the dark at 37°C with 1 × 10^−5^ M ADPM01 for 1 h. The medium was removed and the cells then treated with 250 nM MitoTracker Green (Molecular Probes) or 100 nM ER Tracker Blue (Molecular Probes) for 25 min. Prior to visualisation, excess probe was washed off by rinsing in PBS four times and cells were fixed in 3.7% formaldehyde/PBS. Cells were mounted as above and image analysis was performed using a LSM510 META confocal laser-scanning microscope (Zeiss) equipped with an × 40 numerical aperture 1.0 objective, with a pinhole of 100 *μ*m in diameter being used to capture each image at a resolution of 512 × 512 pixels. MitoTracker Green was excited by a 488 nm argon laser and ER Tracker Blue was excited by a UV laser (364 nm), while ADPM01 was excited by a 543 nm helium neon laser. For simultaneous detection of MitoTracker Green and ADPM01, a 560 nm long pass filter was used in channel 1 (red) for ADPM01, while a 505–530 nm band pass filter was used in channel 2 (green) for MitoTracker Green. For dual detection of ER Tracker Blue and ADPM01, a long pass filter of 560 nm was used in channel 1 (red) for ADPM01 and a 385–470 nm band pass filter was used in channel 2 (blue) for ER Tracker Blue. Using the confocal microscope, single-channel lambda scans were performed (a long pass filter of 560 nm was used in channel 1 (red) for ADPM01), in order to facilitate spectral characterisation of ADPM01 compound within cells. Cell imaged with a lower concentration of ADPM01 (5 × 10^−6^ M) gave similar localisation profiles.

### Colorimetric survival assay

Cells were seeded in 96-well flat-bottomed microtitre plates (5000 cells well^−1^) and incubated at 37°C in a 5% CO_2_ incubator for 24 h. Cells were then incubated with varying concentrations of photosensitiser (1 × 10^−9^ to 1 × 10^−4^ M) in the dark for 3 h at 37°C. The ADPM compound-laden medium was then removed, the cells were washed with PBS and fresh culture medium was added to each well. This was followed by light irradiation with a broad-spectrum red light source (600–750 nm) via a Waldmann PDT1200L light delivery system. For this study, fluences (light exposures) of either 8 or 16 J cm^−2^, at a dose rate of 80 mW cm^−2^, were used. Following irradiation, the cells were incubated for a further 48 h before being assessed for cell viability by a standard MTT assay (Sigma). The subsequent colour change produced by viable cells was quantified using a plate reader (VICTOR^2^ 1420 multilabel HTS counter, Wallac). Potential toxicity attributed to the vehicle, CrEL/1,2-propanediol, was found to be minimal. Every experiment had an internal control, whereby no photosensitiser was administered to the cells and results were expressed as % control, which were irradiated along with the treated cells. The dark toxicity of each compound was also assessed.

For cell viability experiments performed under hypoxic conditions, cells were cultured in a hypoxia chamber as described above. Varying concentrations of ADPM compounds were made using medium equilibrated within the hypoxic environment. After 3 h, the medium containing photosensitiser was removed, the cells were washed with PBS to remove excess compound and fresh, equilibrated medium was added to each well. This was followed by light irradiation under hypoxic conditions with the broad-spectrum red light source as before.

### Flow cytometric assessment of apoptosis

Evaluation of apoptotic and necrotic cell populations was achieved using the ApoTarget Annexin-V FITC Apoptosis kit (BioSource International, Inc.). Cells (2.0 × 10^5^) were seeded into 25 cm^2^ flasks and treated with either ADPM01 or ADPM06 at respective EC_50_ or 10-fold EC_50_ concentration levels for 3 h, followed by irradiation with red light at 16 J cm^−2^, as described above. Subsequently, cells were incubated for 4 and 16 h, with floating and adherent cells being collected at each time point by centrifugation and then stained with Annexin-V and propidium iodide (PI) as per the manufacturer's instructions. The percentage of cells at different phases of cell death, namely nonapoptotic (Annexin-V^−^/PI^−^), early apoptotic (Annexin-V^+^/PI^−^), and necrotic or late-stage apoptotic (Annexin-V^+^/PI^+^), was quantified using a EPICS XL-MCL flow cytometer (Coulter) and analysed using System II software, with at least 5000 cells being analysed for each run.

### Visualisation of apoptosis by immunofluorescence microscopy

Cells (1.5 × 10^4^ cm^−2^) were seeded into chamber slides for 24 h, treated with drug for 3 h and exposed to red light at 16 J cm^−2^, as described above. After 16 h, cells were washed in PBS, fixed on coverslips with 3% formaldehyde in cell culture medium for 20 min at room temperature and washed three times with PBS. The cells were then permeabilised for 10 min with 0.2% Triton X-100 in PBS and washed three times with PBS. Fixed cells were subsequently co-stained with 400 mU ml^−1^ of rhodamine phallodin (Molecular Probes) for 30 min to stain for F-actin and, thereafter, stained with 1 *μ*g ml^−1^ DAPI for 10 min. After being washed thoroughly, cells were mounted and viewed at magnification × 63 with a Zeiss Axioplan imaging microscope, with images being obtained using Zeiss AxioCam at a resolution of 1300 × 1030 pixels.

### Data analysis

Prism (Bioimaging Systems) was used to graph the data obtained from the MTT assays and the uptake/clearance experiments. This program allows nonlinear regression analysis and the generation of sigmoidal dose–response curves. Prism also automatically calculates EC_50_ values. Statistical analysis was performed using the ANOVA test (Prism). Significant differences were detected using the Bonferroni multiple comparison test. *P*-values <0.05 were considered statistically significant.

## RESULTS

### Structure and formulation of ADPM01 and ADPM06

**Figure 1:**
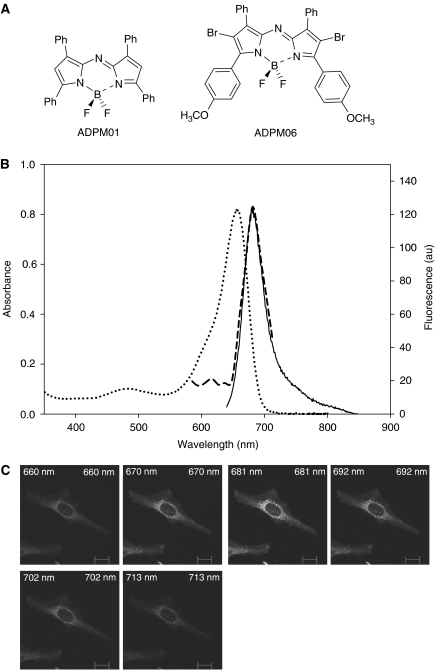
Structural and spectral characteristics of ADPM series. (**A**) Chemical structures of ADPM01 and ADPM06. (**B**) Overlaid UV–Visible spectrum (**^……^**), fluorescent spectrum (**——**) and intracellular fluorescent spectrum (**- - - - -**) of formulated ADPM01. (**C**) Confocal lambda profile (wavelength scan) of ADPM01 in HeLa cells (scale bar indicates 6 *μ*m).

The chemical structure of the ADPM class of photosensitiser is unique and differs significantly from other classes of poly-pyrrole macrocycles (Figure 1A). For drug delivery purposes, defined solutions of ADPM01 and ADPM06 in PBS were generated using the emulsifier CrEL. CrEL is a non-ionic surfactant, frequently used *in vivo* as a delivery agent for poorly water-soluble anti-cancer drugs such as Paclitaxel (Gelderblom *et al*, 2001). New photosensitiser formulations were made immediately prior to use and filtered to ensure homogeneity. Photosensitiser concentration following formulation was determined by UV–Visible spectral analysis of the solutions prior to use. The absorbance spectral characteristics of formulated ADPM01 and ADPM06 are optimal for use in PDT applications, with strong absorbance bands at 658 and 683 nm, respectively (Gorman *et al*, 2004). Analysis of intracellular fluorescence spectra of ADPM01 using confocal laser-scanning microscopy gave almost an identical fluorescence spectrum compared to the aqueous formulated solution, indicating that photosensitiser aggregation is not occurring within cells (Figure 1B and C).

### Time-dependent uptake and clearance of ADMP01

**Figure 2:**
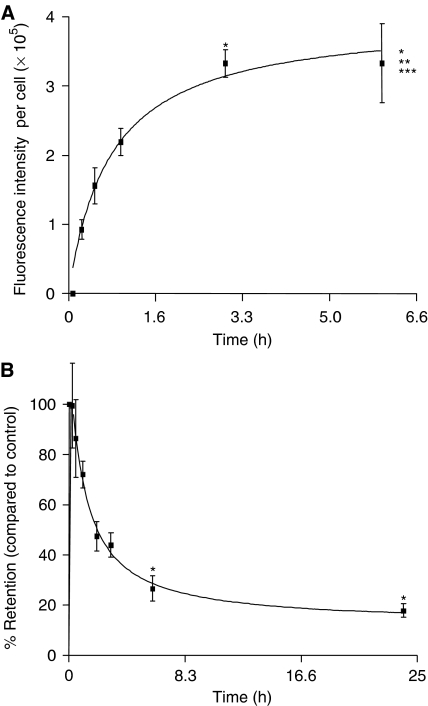
Cellular uptake, clearance and subcellular localisation of ADPM01 in HeLa cells. Cellular uptake (**A**) and clearance (**B**) of ADPM01 in HeLa cells over 6 and 24 h periods, respectively. Each data point represents the mean fluorescent intensity per cell in five fields of view (minimum of 30 cells per field of view, × 20 magnification). Graphs illustrate the average of three independent experiments. Statistics for (**A**), ANOVA: *P*<0.0001, *F*=10.72, Bonferroni multiple comparison test: ^*^P<0.01, compared to 15 min time point; ^**^P<0.05, compared to 30 min time point; ^***^P<0.05, compared to 1 h time point. Statistics for (**B**), ANOVA: *P*=0.0017, *F*=5.46. Bonferroni multiple comparison test: ^*^P<0.05, compared to control, that is, immediately after removal of the drug.

We have previously reported ADPM01 to have a high fluorescence quantum yield (*Φ*_f_=0.34), which is far greater than that observed for ADPM06 (*Φ*_f_=0.1) (Gorman *et al*, 2004). For this reason, ADPM01 was used for all cellular imaging assays. To determine the appropriate conditions for scheduling of light-induced cytotoxicity assays, we determined the uptake and clearance kinetics of ADMP01 in HeLa and MRC5 cells (Figure 2; Supplementary data). Rapid uptake of ADPM01 in HeLa cells was observed over a 6 h period, with >90% being accumulated by 3 h (Figure 2A). A similar rate of uptake was seen in the case of MRC-5 cells (Supplementary data). While clearance of accumulated ADMP01 from HeLa cells was also rapid with only 42% retention at 3 h post-withdrawal (Figure 2B), MRC-5 cells displayed slower release kinetics of 74% retention, 3 h post-withdrawal (Supplementary data).

### Subcellular localisation of ADPM01

**Figure 3:**
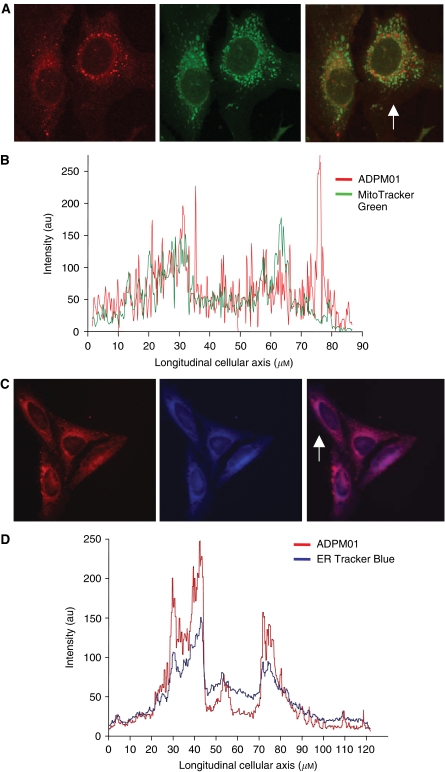
Subcellular localisation of ADPM01. (**A**) Co-incubation of ADMP01 and MitoTracker Green (mitochondria-specific probe) in HeLa cells. ADPM01 (red), MitoTracker Green (green) and overlay images shown from left to right (arrow indicates the analysed cell). (**B**) Longitudinal transcellular overlay of fluorescence intensity for ADMP01 and MitoTracker Green. (**C**) Co-incubation of ADPM01 and ER Tracker Blue (endoplasmic reticulum-specific probe) in HeLa cells. ADPM01 (red), ER Tracker Blue (blue) and overlay images shown from left to right (arrow indicated the analysed cell). (**D**) Longitudinal transcellular overlay of fluorescence intensity for ADPM01 and ER Tracker Blue.

It has previously been shown that ADPM01 does not localise to the nucleus (Gorman *et al*, 2004). Here, we further examined the subcellular localisation of ADPM01 in HeLa cells via confocal microscopy using dual staining techniques. ADPM01 displayed partial co-localisation with mitochondrial regions (Figure 3A). A more striking co-localisation was shown for ADPM01 and the endoplasmic reticulum (Figure 3C). Image analysis was carried out for the localisation profile of the organelle-specific probes and ADPM01 along the longitudinal axis of single cells. Use of MitoTracker Green (a mitochondria-specific probe) suggested a relatively weak accumulation of ADPM01 in mitochondria, which is reflected in the poor overlap of a number of fluorescent peaks in the image analysis (Figure 3B). In comparison, image evaluation of co-localisation between ER Tracker Blue and ADPM01 revealed a comparable pattern of localisation within the cells, with an almost identical overlap between the transcellular longitudinal fluorescence localisation profiles (Figure 3D). These data appear to strongly indicate the localisation of ADPM01 within the endoplasmic reticulum; however, due to their close perinuclear proximity, additional localisation within the Golgi apparatus cannot be ruled out.

### Cytotoxic potential of ADPM01 and ADPM06

**Figure 4:**
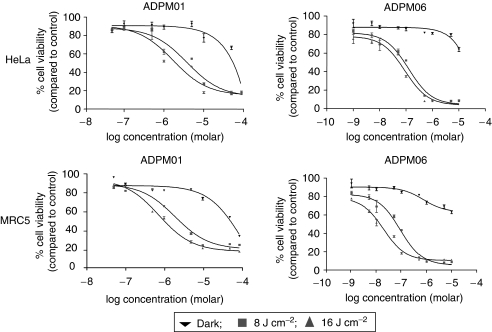
Azadipyrromethene-mediated cytotoxicity. Representative graphs illustrating the light-induced cytotoxicity of ADPM01 and ADPM06 in HeLa and MRC5 cells for three light doses, 0 J cm^−2^ (dark) (▾), 8 J cm^−2^ (▪) and 16 J cm^−2^ (▴) in normoxic conditions.

The kinetics of ADPM01 accumulation indicated an optimal incubation time of 3 h prior to irradiation. We examined the effects of ADPM01 and ADPM06 against a broad range of cell lines derived from a number of different tissue types, including lung, breast, ovary, colon and cervix (Figure 4; Table 1). In all cell lines examined, ADPM01 and ADPM06 displayed potent cytotoxic activity following photo-irradiation. Both compounds showed light dose-dependent cytotoxicity, with greater cell killing observed at 16 J cm^−2^ as compared with 8 J cm^−2^. EC_50_ values for each compound varied between the different cell lines examined (ADPM01, 2.8 × 10^−6^–3.4 × 10^−5^ M for 8 J cm^−2^ and 2.6 × 10^−7^–2.2 × 10^−5^ M for 16 J cm^−2^; APDM06, 3.7 × 10^−8^–5.4 × 10^−6^ M for 8 J cm^−2^ and 1.4 × 10^−8^–6.3 × 10^−7^ M for 16 J cm^−2^); however, no apparent bias in terms of activity towards particular cell types was observed. Notably, the A2780 cell line and its cisplatin-resistant derivative, A2780cp70, displayed no difference with respect to effectiveness of either ADPM01 or ADPM06 (Table 1). In a similar vein, no distinction was seen between SW480 (colon carcinoma) and SW620 (metastatic variant of SW480) cells in this respect. The consistently improved efficacy of ADPM06 over ADPM01 is attributed to the bromine substituents having a heavy-atom effect, which results in greater efficiency of singlet oxygen generation (Gorman *et al*, 2004).

### ADMP06 retains activity in hypoxic conditions

**Figure 5:**
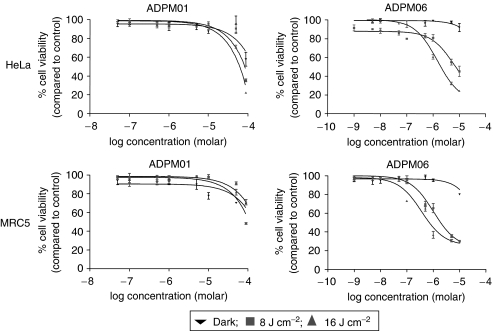
Azadipyrromethene-mediated cytotoxicity. Representative graphs illustrating the light-induced cytotoxicity of ADPM01 and ADPM06 in HeLa and MRC5 cells for three light doses, 0 J cm^−2^ (dark) (▾), 8 J cm^−2^ (▪) and 16 J cm^−2^ (▴) in hypoxic (1% oxygen) conditions.

The effect of hypoxia on ADPM-mediated PDT was assessed in HeLa and MRC5 cells (Figure 5; Table 2). In both cell lines, the efficacy of ADPM01 was completely ablated at a 1% oxygen level. In contrast, ADPM06 displays only a partial reduction in light-induced activity in hypoxic as compared to normoxic conditions. In the low-oxygen environment with a light dose of 16 J cm^−2^, ADPM06 retains considerable efficacy, with EC_50_ values of 1.5 and 1.6 × 10^−6^ M for HeLa and MRC5 cells, respectively (Table 2).

### ADPM01 and APDM06 induce apoptosis

**Figure 6:**
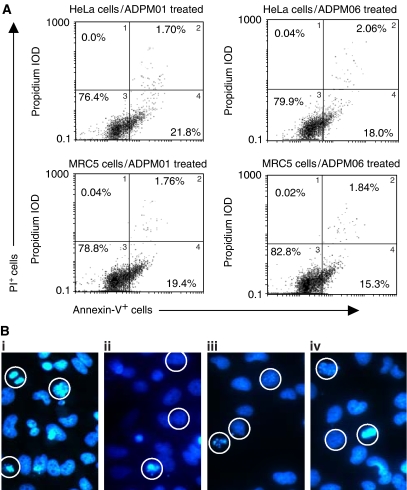
Azadipyrromethene-mediated induction of apoptosis. (**A**) Induction of apoptotic cell death in HeLa and MRC5 cells by ADPM01 and ADPM06 at EC_50_ concentration level, 16 h post-irradiation. Cells were stained with PI and Annexin V and subsequently analysed by flow cytometry. Percentages for each quadrant are shown. (**B**) Immunofluorescence assay images of ADPM-treated HeLa and MRC5 cells showing areas of chromatin condensation and apoptotic body formation (circled cells) at 16 h time point post-irradiation (F-actin stain not shown).

Flow-cytometric analysis of HeLa and MRC5 cells exposed to either ADPM01 or ADPM06 at respective EC_50_ values showed that apoptosis had commenced at 4 h post-irradiation (Supplementary data). At 16 h post-irradiation, the predominant mode of cell death was apoptosis for both photosensitisers (Figure 6A). The induction of apoptosis was confirmed by immunofluorescence microscopy, which showed chromatin condensation patterns in each case, as well as apparent apoptotic bodies (Figure 6B, circled cells). Exposure of cells to a higher concentration of ten-fold EC_50_ level resulted in a marked increased necrotic/late apoptotic response being observed (Supplementary data).

## DISCUSSION

PDT was first approved for clinical use in Canada in 1993, with Photofrin being used for the treatment of bladder cancer. Subsequently, approval has been granted in various countries for early- and late-stage lung cancers, oesophageal, gastric and cervical cancers. The recognised drawbacks of Photofrin are that it is an undefined, complex mixture of haematoporphyrin derivatives, displays poor photophysical characteristics and has a propensity to cause long-lasting skin sensitivity due to retention in skin tissue (Sibata *et al*, 2001). Uptake and clearance rates of ADPM01 suggest that this family of photosensitisers may be clinically beneficial. In more detail, ADPM01 is rapidly taken into tumour cells, with >90% uptake achieved within 3 h. The kinetics of cellular uptake of ADPM01 are similar to those seen with other photosensitisers (Khanum and Jain, 1997; Gupta *et al*, 2003). Of key importance, ADPM01 is also rapidly cleared from tumour cells. Interestingly, dark toxicity of ADPM01 was found to be limited in MRC5 cells compared to that seen in HeLa cells, even despite slower release kinetics in the former cell type.

Since singlet oxygen has a short half-life and can only travel minimal distances while still retaining activity, the intracellular distribution of photosensitisers is an important factor in determining the efficacy of PDT (Peng *et al*, 1996). In recent years, various photosensitisers have been shown to localise to the mitochondria, lysosomes, nuclei and plasma membranes. It has previously been demonstrated that localisation to the mitochondria and endoplasmic reticulum may be beneficial in PDT terms (Morgan and Oseroff, 2001; Teiten *et al*, 2003). We have shown that ADPM01 localises to the endoplasmic reticulum and, to a lesser extent, the mitochondria of *in vitro* cultured cells. The mitochondria play a key role in energy production and calcium homeostasis within cells. The rapid growth of tumour cells is highly energy-dependent, so disruption to mitochondrial function could be a significant therapeutic target. Disruption of endoplasmic reticulum functions relating to protein folding and calcium storage could also be key to a PDT effect.

In the present study, we have shown that ADPM01 and ADMP06 potently inhibit the viability of a broad range of cell types, with no apparent bias towards tumour cells of any particular tissue origin. Moreover, isogenic drug-resistant and metastatic derivatives displayed sensitivities to both ADPM01 and ADMP06 comparable to that shown by their parental counterparts. Overall, this provides strong support for the application of ADPM-mediated PDT to a wide variety of cell types *in vitro* and tumours *in vivo*.

Cell death induced by PDT has been shown to be the result of either apoptosis and/or necrosis (Oleinick *et al*, 2002), depending on the cell type, intracellular localisation and concentration of the photosensitiser (Kessel and Luo, 1998), as well as incubation conditions and the light dose used (Dellinger, 1996). We have shown that ADPM01 and ADPM06 both induce apoptosis when the sensitiser is administered at EC_50_ concentration levels. It still remains to be determined which apoptotic pathways are being triggered by ADPM-mediated PDT. However, a combination of mitochondrial and endoplasmic reticulum stress-induced apoptotic pathways would be reasonable suggestions based on subcellular localisation data (Grebeòová *et al*, 2003). It is evident, however, that induction of cell death by ADPM photosensitisers is rapid.

PDT requires molecular oxygen during light irradiation to generate reactive oxygen species, such as singlet oxygen. In addition to pre-existing tumour hypoxia, the process of PDT itself induces hypoxia, as the generation of reactive oxygen species is an oxygen-consuming process (Foster *et al*, 1991). PDT also causes destruction of the microcirculation, further increasing hypoxia within minutes (Reed *et al*, 1989). It is well established that hypoxia can abolish the effect of PDT using haematoporphyrin derivatives (Mitchell *et al*, 1985; Moan and Sommer, 1985). In normal tissues, the oxygen tension is usually in the range of 5–10%, whereas in tumours values of 0–5% are more common (Wyld *et al*, 1998). Tumour hypoxia can, therefore, severely hamper PDT efficacy. Here, we show that hypoxia completely abolished the efficacy of the less active agent, ADPM01. Encouragingly, the efficacy of ADPM06, which has been shown to be a more effective singlet oxygen generator than ADPM01, remains acceptable even under hypoxic conditions, implying a more efficient use of the limited supply of oxygen.

Taken together, our data show the ADPM family of compounds to be a promising new class of photosensitisers, with potent activity against a broad range of tumour cell types. Indeed, ADPMs appear to be more effective, especially in terms of tumour cell destruction, than many photosensitizing agents currently available.

## Figures and Tables

**Table 1:** ADPM-mediated cytotoxicity^a^^,^^b^

		**ADPM01**				**ADPM06**	
**Cell line**	**Tissue origin**	**EC_50_ (M) (dark)**	**EC_50_ (M) (8 J cm^−2^)**	**EC_50_ (M) (16 J cm^−2^)**	**EC_50_ (M) (dark)**	**EC_50_ (M) (8 J cm^−2^)**	**EC_50_ (M) (16 J cm^−2^)**
HeLa	Cervix	2.7 × 10^−5^ (±1.0)	2.8 × 10^−6^ (±0.7)	9.2 × 10^−7^ (±1.0)	>10^−4^	6.3 × 10^−8^ (±2.0)	4.1 × 10^−8^ (±3.0)
MRC-5	Lung	>10^−4^	3.1 × 10^−6^ (±3.0)	5.0 × 10^−7^ (±1.0)	>10^−4^	3.7 × 10^−8^ (±0.3)	1.4 × 10^−8^ (±0.1)
HT-1080	Connective tissue from hip socket	>10^−4^	3.4 × 10^−5^ (±0.9)	2.2 × 10^−5^ (±4.0)	4.4 × 10^−6^ (±4.0)	7.9 × 10^−8^ (±1.0)	3.7 × 10^−8^ (±5.0)
T47-D	Breast	>10^−4^	5.6 × 10^−6^ (±0.5)	5.3 × 10^−6^ (±1.9)	>10^−4^	1.6 × 10^−6^ (±0.8)	6.3 × 10^−7^ (±3.3)
MCF-7	Breast	>10^−4^	3.4 × 10^−6^ (±1.7)	1.9 × 10^−6^ (±4.7)	>10^−4^	1.2 × 10^−7^ (±3.1)	7.5 × 10^−8^ (±2.1)
MDA-MB-231	Breast	1.2 × 10^−5^ (±0.5)	5.6 × 10^−6^ (±3.0)	2.6 × 10^−7^ (±0.5)	>10^−4^	1.4 × 10^−7^ (±1.9)	7.5 × 10^−8^ (±1.2)
SW480	Colon	>10^−4^	7.6 × 10^−6^ (±0.9)	8.0 × 10^−7^ (±0.7)	>10^−4^	5.4 × 10^−6^ (±1.0)	4.2 × 10^−8^ (±0.2)
SW620	Colon	>10^−4^	5.6 × 10^−6^ (±0.7)	2.8 × 10^−6^ (±0.7)	>10^−4^	7.5 × 10^−8^ (±2.9)	6.0 × 10^−8^ (±1.1)
A2780	Ovary	>10^−4^	5.1 × 10^−6^ (±5.1)	4.5 × 10^−6^ (±1.2)	>10^−4^	1.0 × 10^−7^ (±0.7)	3.5 × 10^−8^ (±4.2)
A2780cp70	Ovary	>10^−4^	6.8 × 10^−6^ (±3.2)	2.8 × 10^−6^ (±2.8)	>10^−4^	6.2 × 10^−8^ (±3.1)	2.7 × 10^−8^ (±1.2)

a Cytotoxic potential of compounds assessed *in vitro* using a standard MTT assay. Cells were incubated with PDT agent for 3 h prior to irradiation with 0, 8 or 16 J cm^−2^.

b Mean and standard deviation (in parenthesis) of triplicate determinations.

**Table 2:** Effect of hypoxia on ADPM-mediated cytotoxicity^a^

		**ADPM01**			**ADPM06**		
	**Oxygen conditions**	**EC_50_ (M) (dark)**	**EC_50_ (M) (8 J cm^−2^)**	**EC_50_ (M) (16 J cm^−2^)**	**EC_50_ (M) (dark)**	**EC_50_ (M) (8 J cm^−2^)**	**EC_50_ (M) (16 J cm^−2^)**
HeLa	Normoxia^b^	2.7 × 10^−5^ (±1.0)	2.8 × 10^−6^ (±0.7)	9.2 × 10^−7^ (±1.0)	>10^−4^	6.3 × 10^−8^ (±2.0)	4.1 × 10^−8^ (±3.0)
	Hypoxia^c^	>10^−4^	>10^−4^	>10^−4^	>10^−4^	6.1 × 10^−6^ (±2.2)	1.5 × 10^−6^ (±2.0)
MRC5	Normoxia^b^	>10^−4^	3.1 × 10^−6^ (±3.0)	5.0 × 10^−7^ (±1.0)	>10^−4^	3.7 × 10^−8^ (±0.3)	1.4 × 10^−8^ (±0.1)
	Hypoxia^c^	>10^−4^	>10^−4^	>10^−4^	>10^−4^	7.1 × 10^−6^ (±2.6)	1.6 × 10^−6^ (±1.8)

a Cells were grown under either normoxic or hypoxic conditions prior to PDT, with cell viability assessed by a standard MTT assay.

b Normoxia=21% oxygen.

c Hypoxia=1% oxygen.
