# Supplementary material for: A potent nonporphyrin class of photodynamic therapeutic agent: cellular localisation, cytotoxic potential and influence of hypoxia
Source: Br J Cancer. 2005 Apr 19;92(9):1702–10. doi: 10.1038/sj.bjc.6602527 (PMC2362044; doi:10.1038/sj.bjc.6602527)

Supplemental Fig. 1: Cellular uptake, clearance and sub-cellular localization of ADPM01 in MRC5 cells. Cellular uptake (**A**) and clearance (**B**) of ADPM01 in MRC5 cells over 6 and 24 hr periods, respectively. Each data point represents the mean fluorescent intensity/cell in 5 fields of view (minimum of 30 cells/field of view, x20 magnification). Graphs illustrate the average of three independent experiments. DAPI nuclear stain was used to count cell number/field (data not shown). Statistics for **A**, ANOVA: $p < 0.0001$ F=10.72, Bonferroni Multiple Comparison test: * $p < 0.01$, compared to 15 min time point; ** $p < 0.05$, compared to 30 min time point; *** $p < 0.05$, compared to 1 hr time point. Statistics for **B**, ANOVA: $p = 0.0017$, $F = 5.46$. Bonferroni Multiple Comparison test: * $p < 0.05$, compared to control, i.e., immediately after removal of the drug.

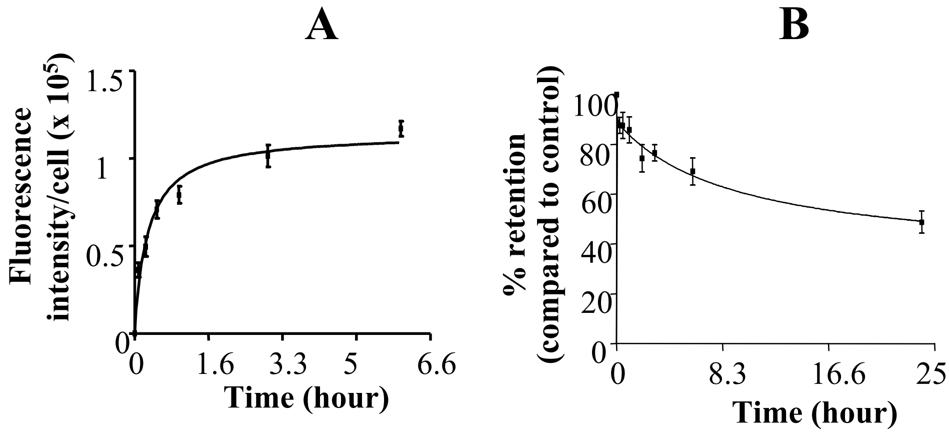

Supplemental Fig. 2: ADPM-mediated induction of apoptosis. Induction of apoptotic cell death in HeLa and MRC5 cells by ADPM01 and ADPM06 at EC-50 concentration level, 4 h post-irradiation. Cells were stained with PI and Annexin V and subsequently analyzed by flow cytometry. Percentages for each quadrant are shown.

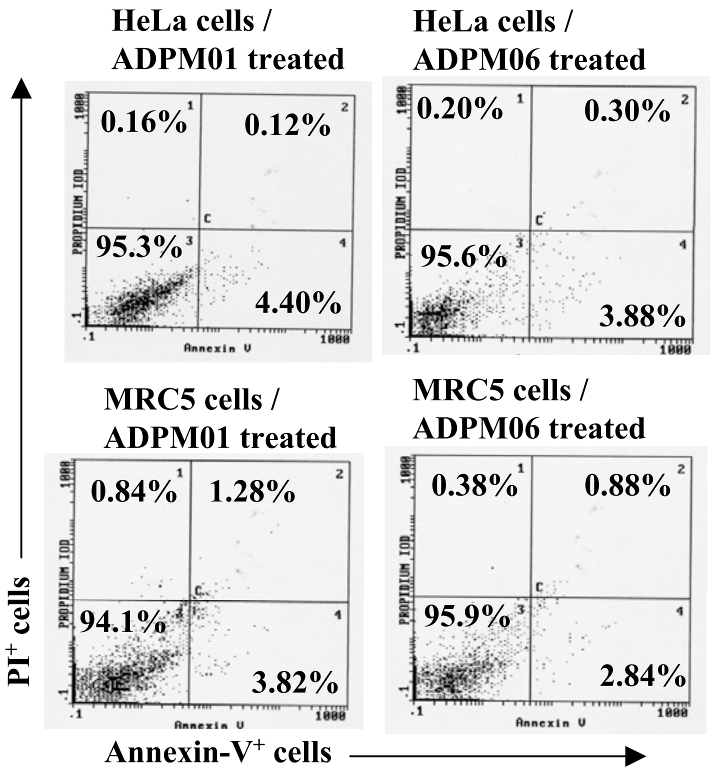

Supplemental Fig. 3: ADPM-mediated induction of apoptosis at 10 fold EC-50 level. Induction of apoptotic cell death in HeLa and MRC5 cells by ADPM01 and ADPM06 at 10-fold EC-50 concentration level, 16 h post-irradiation. Cells were stained with PI and Annexin V and subsequently analyzed by flow cytometry. Percentages for each quadrant are shown

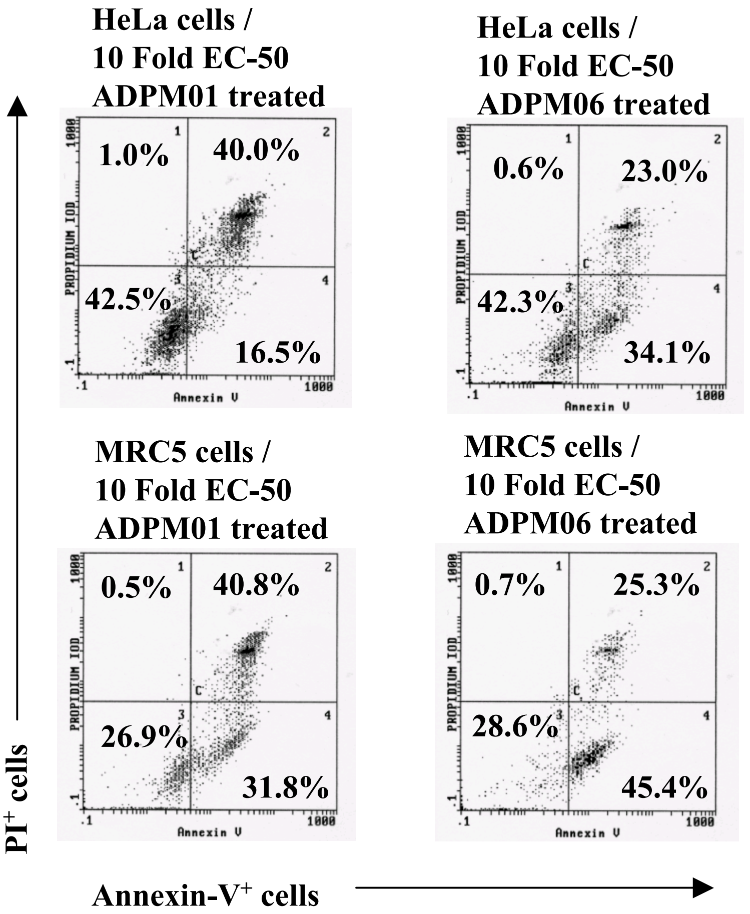

Supplement: Supplementary data [file 92-6602527x1.pdf]
